# Evidence for genetic association of *TBX21* and *IFNG* with systemic lupus erythematosus in a Chinese Han population

**DOI:** 10.1038/srep22081

**Published:** 2016-02-26

**Authors:** Rui-Xue Leng, Hai-Feng Pan, Juan Liu, Xiao-Ke Yang, Chao Zhang, Sha-Sha Tao, De-Guang Wang, Xiao-Mei Li, Xiang-Pei Li, Wanling Yang, Dong-Qing Ye

**Affiliations:** 1 Department of Epidemiology and Biostatistics, School of Public Health, Anhui Medical University, Hefei, Anhui, China; 2 Anhui Provincial Laboratory of Population Health and Major Disease Screening and Diagnosis, Anhui Medical University, Hefei, Anhui, China; 3 Department of Nephrology, The Second Affiliated Hospital of Anhui Medical University, Hefei, Anhui, China; 4 Department of Rheumatology and Immunology, Anhui Provincial Hospital, Affiliated to Anhui Medical University, Hefei, Anhui, China; 5 Department of Paediatrics and Adolescent Medicine, University of Hong Kong, Hong Kong, China

## Abstract

*TBX21* recode T-bet which is an important transcription factor that drives the Th1 immune response primarily by promoting expression of the interferon-gamma (*IFNG*) gene. Recent studies have shown that genetic variants in *TBX21* and *IFNG* are connected with risk of systemic lupus erythematosus (SLE). The aim of the present study was to replicate these genetic associations with SLE in Anhui Chinese population. Genotyping of 3 variants (rs4794067 in *TBX21*, rs2069705 and rs2069718 in *IFNG*) was performed. A total of 3732 subjects were included in the final analysis. The study only identified the association of rs2069705 with SLE susceptibility (T vs. C: odds ratio [OR] = 1.12, 95% confidence interval [CI] = 1.00–1.26, *P* = 0.046). Combined analysis with Hong Kong GWAS showed that the OR for rs2069705 was 1.10 (95% CI: 1.01–1.21, *P* = 0.027). Further pooled analysis with Korean populations involving 10498 subjects showed a more significant association between rs2069705 and SLE (T vs. C: OR = 1.11, 95%CI = 1.04–1.19, *P* = 0.002; TT + TC vs. CC: OR = 1.11, 95%CI = 1.02–1.21, *P* = 0.012; TT vs. TC + CC: OR = 1.28, 95%CI = 1.07–1.54, *P* = 0.008; TT vs. CC: OR = 1.33, 95%CI = 1.10–1.60, *P* = 0.003). In addition, we also identified a significant genetic interaction between rs2069705 and rs4794067 in Anhui Chinese population. Our study suggests that *IFNG* and *IFNG-TBX21* interaction are involved in SLE susceptibility.

Systemic lupus erythematosus (SLE) is a prototypic, systemic, autoimmune disease, characterized by autoantibody production and systemic inflammation. Numerous studies have proved that there was a strong genetic susceptibility and a multifactorial aetiology underlying SLE, in which gene–gene interactions may jointly affect the onset of the disease^1^,^2^,^3^.

The transcription factor T-bet (*TBX21*) is the master regulator of Th1 cell fate primarily by promoting expression of the interferon-gamma (*IFNG*) gene. IFNG is a type II interferon playing diverse roles in innate and adaptive immune systems. Elevated expression of *IFNG* has been found in peripheral blood T cells of SLE patients^4^. Furthermore, in murine models of lupus, deletion of *Ifng* leads to an improvement of lupus-like symptoms^5^. Available data has shown that *TBX21* polymorphisms are associated with risk of several autoimmune diseases including rheumatoid arthritis (RA)^6^ and systemic sclerosis (SSc)^7^. It has been demonstrated that the C allele of rs4794067 in the *TBX21* promoter is associated with a significant decrease in T-bet and IFNG production by stimulated human lymphocytes from healthy individuals^8^,^9^. A previous study reported that the frequency of C allele of rs4794067 was significantly lower in SLE patients than in controls in Chongqing Chinese Han population. It should be noted that the cohort in this study were too small (only 248 cases and 261 controls were included) to have enough power to detect real association^10^. Previous study also reported that several *IFNG* polymorphisms were associated with susceptibility to SLE in unrelated Korean subjects^11^,^12^. However, no replication study has evaluated for this relationship of *IFNG* in other ancestries. It is also unknown whether there is a genetic interaction between *TBX21* and *IFNG* given their functional association. We therefore investigated possible associations between above genes and SLE in a Chinese Han population with relatively larger samples (3732 subjects).

## Results

### Polymorphisms in *IFNG* and *TBX21* with SLE risk

A total of 3732 subjects (1466 SLE patients and 2266 controls) were included in the final analysis. The mean ± SD age of patients with SLE was 36.2 ± 12.7 years, and 91.5% were women. The mean ± SD age of the control subjects was 36.9 ± 14.7 years, and 84.5% were women. No single-nucleotide polymorphism (SNP) deviated from HWE (*P* ≥ 0.506). The result of association for each SNP is shown in Table 1. In this study, we only identified that the T allele of rs2069705 in *IFNG* promoter was marginally associated with SLE susceptibility in Anhui Chinese Han population (T vs. C: odds ratio [OR] = 1.12, 95% confidence interval [CI] = 1.00–1.26, *P* = 0.046), but did not survive bonferroni correction. Meanwhile, only a similar trend for rs2069705 was observed in Hong Kong genome-wide association studies (GWAS) (T vs. C: OR = 1.08, 95% CI: 0.94–1.24). The pooled OR for rs2069705 in the two Chinese populations (including 2072 cases and 4432 controls) was 1.10 (95% CI: 1.01–1.21, *P* = 0.027) (Table 2). Given the similar association of rs2069705 with SLE observed in Korean populations, we carried out a meta-analysis of all cases and controls (a total of 10498 subjects including 3599 patients with SLE and 6899 healthy controls) to maximize the number of samples for the association analysis. The combined analysis showed a significant association between rs2069705 and SLE (T vs. C: OR = 1.11, 95%CI = 1.04–1.19, *P* = 0.002; TT + TC vs. CC: OR = 1.11, 95%CI = 1.02–1.21, *P* = 0.012; TT vs. TC + CC: OR = 1.28, 95%CI = 1.07–1.54, *P* = 0.008; TT vs. CC: OR = 1.33, 95%CI = 1.10–1.60, *P* = 0.003). We further performed subgroup analysis according to ethnicity (Chinese or Korean) and genotyping method. The data showed a significant and consistent trend toward increased risk of SLE in different subgroups (Table 2). In contrast with previous studies, significant association with SLE susceptibility was not observed for rs4794067 (C vs. T: *P* = 0.647) and rs2069718 (C vs. T: *P* = 0.707) in Anhui Chinese population (Table 1).

### *IFNG* and *TBX21* interaction with SLE susceptibility

Significant multiplicative interaction was detected between rs4794067 and rs2069705 in both codominant and dominant model. After adjusting for age and sex, this association was still statistically significant (Table 3). In contrast, significant interaction was not observed for rs4794067-rs2069718 combination. Because the numbers for some genotypes were small, and in most cases dominant models showed the better fit for association, we considered the dominant model in a further additive interaction analysis stage. The following analysis also identified a significant interaction (the relative excess risk due to interaction [RERI] = 0.66, 95% CI: 0.26–1.07) between rs4794067 and rs2069705 with controlling for age and sex. Similar to the finding of multiplicative interaction, additive interaction analysis between rs4794067 and rs2069718 showed no statistically significant association with SLE susceptibility (Table 3).

## Discussion

Kim, *et al.* reported that rs2069705 in *IFNG* promoter was most significantly associated with SLE susceptibility in Korean subjects^11^. However, there are no replication studies indicating an association between *IFNG* gene and SLE in Chinese population. Our present study revealed that the rs2069705 was associated with SLE in Anhui Chinese Han population. It should be noted that the odds ratio and the significance level for rs2069705 is marginal (*P* = 0.046). Thus, this association may be false positive. To confirm this marginal effect, a meta-analysis by using four studies was conducted to produce a more comprehensive result. The data showed that the SNP was significantly associated with SLE susceptibility in Asians although stratification by race reveals this genetic association is found more in Korean than in Chinese population. These findings suggest that the SNP (rs2069705) in *IFNG* promoter may be a common variant in Asian populations, and thus might improve our understanding of the pathogenesis of SLE and provide new targets for therapeutic interventions. In contrast, our replication study suggests that rs4794067 in *TBX21* promoter and rs2069718 in *IFNG* intron are not involved in SLE susceptibility in Chinese Han population. The heterogeneity was probably due to difference in sample size, genotyping method or source of controls^10^,^11^. Therefore, future researches are still required to confirm these findings and investigate other SNPs among these genes in different ancestries.

To test for possible gene–gene interactions, we performed an analysis using multiplicative and additive interaction scales. The identified interaction between *TBX21* (rs4794067) and *IFNG* (rs2069705) may be biologic. Firstly, the transcription factor T-bet (*TBX21*) is the master regulator of Th1 immune response primarily by promoting expression of the *IFNG*. This functional link suggests both *TBX21* and *IFNG* can participate in the same causal mechanism. Secondly, available data has suggests that the C allele rs4794067 in *TBX21* promoter is a functional SNP and is associated with decreased T-bet expression levels^8^. Mechanism analysis further showed that the polymorphism is associated with decreased IFNG production by primary human lymphocytes from healthy participants^9^. Although the significantly associated SNP (rs2069705) in *IFNG* promoter has not been examined for its effects on *IFNG* expression levels, a SNP (rs2430561) in intron of *IFNG* has been reported to affect the *IFNG* expression level^11^,^13^,^14^. Thirdly, elevated expression levels of *IFNG* are involved in the pathogenesis of SLE in both human disease and murine models^4^,^5^. Therefore, such genetic interaction may imply that *TBX21* can act as a collaborator with *IFNG* to contribute to risk of SLE although the polymorphism in *TBX21* alone does not increase susceptibility of the disease. However, further researches in this field are warranted to confirm this genetic interaction by using both statistical methods and functional studies.

In summary, our study identified a genetic variant in *IFNG* that is significantly associated with SLE risk in Chinese populations. We also provided evidence that gene–gene interaction between *TBX21* and *IFNG* may jointly affect the susceptibility of SLE. These findings highlight Th1 cell response as an important contributor to the pathogenesis of SLE.

## Materials and Methods

### Patients and controls

Patients with SLE were recruited from the First Affiliated Hospital of Anhui Medical University and Anhui Provincial Hospital (Affiliated to Anhui Medical University). The diagnosis of SLE was based on the presence of the combination of at least four criteria of 1997 American College of Rheumatology (ACR) revised criteria for the classification of SLE^15^,^16^. The healthy controls were matched to the patients geographically and ethnically. Informed consent was obtained from all patients and healthy control subjects. The study was approved by the medical ethics committee of Anhui Medical University. Methods were carried out in accordance with the approved guidelines.

### Genotyping

We genotyped rs2069705 in the *IFNG* promoter and rs2069718 in the *IFNG* intron, which were associated with risk of SLE in Korean population^11^. In addition, the functional SNP (rs4794067) in the *TBX21* promote was selected for the current study^8^,^9^,^10^. The genotyping was conducted using Sequenom Massarray system (Sequenom Inc., San Diego, CA, USA). The genotype success rates for the three SNPs were higher than 98%. Only those individuals with 100% genotype success for all markers were included for final analysis.

### Data analysis

The genotype frequencies of the SNPs were tested for Hardy-Weinberg equilibrium in control subjects. Disease associations were analyzed by chi-square test. It should be noted that a total of 22 SNPs were genotyped by the Sequenom Massarray system at the same time. These genotyped SNPs can be mapped into multiple signaling pathways, but only the 3 SNPs (rs2069705, rs2069718 and rs4794067) are directly involved in immune response of Th1 cells. Although prior data have shown that genetic variants in *TBX21* and *IFNG* are connected with risk of SLE in Asians^10^,^11^,^12^. For multiple-testing correction, bonferroni adjustment was still applied and the statistical significance level was set at α = 0.0023 (0.05/22). Association was considered significant for *P* < 0.0023 but marginal when 0.0023 ≤ *P* ≤ 0.05.

The multiplicative gene-gene interactions were estimated using multiple logistic regression models. For each individual, key variables were defined as a binary variable indicating case–control status, with SNP variables ranging from 0 to 2 indicating the number of risk alleles in an individual subject (codominant model). Additionally, dominant model were also tested. For each SNP pair, a logistic regression model was built to predict case–control status (dependent variable) based on the indicator variables (sex and age) and the 2 SNP variables (independent variable), for a total of 4 variables and an intercept^2^,^17^.

For meta-analysis, genotyped data of rs2069705 in three independent studies was available. These data sources included the Anhui study, two Korean studies by Kim, *et al.*^11^,^12^. For archiving a lager statistical power, genotyped information of the SNP from Hong Kong GWAS was extracted (the data was provided by Dr. Wanling Yang, University of Hong Kong) for the pooled analysis^18^. The genotype distribution of rs2069705 in cases from Hong Kong GWAS for CC/TC/TT is 308/249/49, and in controls is 1166/826/174. The pooled estimate of OR and *P* value were obtained by a random-effects (DerSimonian–Laird) or a fixed-effects model (Mantel–Haenszel) in the presence or absence of heterogeneity, respectively. The heterogeneity was assessed by Cochrane’s Q test and the I-square statistic^19^,^20^. In this study, the combined effect sizes were calculated by fixed-effects meta-analysis when p values in Q-statistic ≥ 0.095.

The attributable proportion due to interaction (AP) and RERI were used to test additive gene-gene interactions. If there is no biologic interaction, RERI and AP are equal to 0 2,17. A detailed calculation method of additive interaction including definition of two indicator variables, an STATA program, and a calculator in Excel (available at http://www.epinet.se) was described by Andersson, *et al.*^21^. The STATA program delivered estimates of the required parameters together with the covariance matrix, which are used in calculation of the interaction measures in the Excel calculator. The confounding factors including sex and age were controlled for additive interaction analysis.

Statistical analysis was performed using SPSS13.0 software (SPSS Inc., Chicago, IL, USA) and Stata version 12.0 (Stata Corp, College Station, TX, USA).

## Additional Information

**How to cite this article**: Leng, R.-X. *et al.* Evidence for genetic association of *TBX21* and *IFNG* with systemic lupus erythematosus in a Chinese Han population. *Sci. Rep.* **6**, 22081; doi: 10.1038/srep22081 (2016).

## Figures and Tables

**Table 1:** Genotype characteristics of each single nucleotide polymorphism.

Gene	SNP	Genotype	SLE(1466)	Controls(2266)	HWE	Cases versus controls	
		/Allele	n (%)	n (%)	*P*exact	OR (95% CI)	*P* value
*TBX21*	rs4794067	TT	1118(0.76)	1732(0.76)			
		TC	314(0.21)	494(0.22)			
		CC	34(0.02)	40(0.02)	0.506	1.32(0.83–2.09)*	0.243
		T	2550 (0.87)	3958(0.87)			
		C	382 (0.13)	574(0.13)		1.03(0.90–1.19)	0.647
*IFNG*	rs2069705	CC	885(0.60)	1428(0.63)			
		TC	501(0.34)	742(0.33)			
		TT	80(0.05)	96(0.04)	1.000	1.35(0.99–1.83)#	0.059
		C	2271(0.77)	3598(0.79)			
		T	661(0.23)	934(0.21)		1.12(1.00–1.26)	0.046
*IFNG*	rs2069718	TT	1104(0.75)	1726(0.76)			
		TC	340(0.23)	500(0.22)			
		CC	22(0.02)	40(0.02)	0.572	0.86(0.51–1.46)*	0.573
		T	2548(0.87)	3952(0.87)			
		C	384(0.13)	580(0.13)		1.03(0.89–1.18)	0.707

n, number; SNP, single nucleotide polymorphisms; OR, odds ratio; CI, confidence interval; HWE, Hardy-Weinberg Equilibrium; SLE, systemic lupus erythematosus.

^*^CC versus TT in cases versus controls.

^#^TT versus CC in cases versus controls.

**Table 2:** Meta-analysis of the association between rs2069705 and SLE risk in Asian populations.

Groups/Comparisons	Test of association		Methods	Test of heterogeneity	
	OR (95% CI)	*P*-value		*I*^*2*^	*P*-value
A. Four studies					
T versus C	1.11(1.04–1.19)	0.002	M-H	0.0%	0.481
TT + TC versus CC	1.11(1.02–1.21)	0.012	M-H	0.0%	0.716
TT versus TC + CC	1.28(1.07–1.54)	0.008	M-H	52.8%	0.095
TT versus CC	1.33(1.10–1.60)	0.003	M-H	49.4%	0.115
B. Three studies*					
T versus C	1.13(1.04–1.22)	0.004	M-H	9.5%	0.331
TT + TC versus CC	1.11(1.01–1.22)	0.032	M-H	0.0%	0.515
TT versus TC + CC	1.43(1.15–1.78)	0.002	M-H	41.8%	0.179
TT versus CC	1.46(1.17–1.83)	0.001	M-H	45.2%	0.161
C. Two Chinese studies					
T versus C	1.10(1.01–1.21)	0.027	M-H	0.0%	0.678
TT + TC versus CC	1.12(1.01–1.25)	0.037	M-H	0.0%	0.942
TT versus TC + CC	1.16(0.93–1.45)	0.198	M-H	21.6%	0.259
TT versus CC	1.21(0.96–1.52)	0.099	M-H	0.0%	0.322
D. Two Korean studies					
T versus C	1.13(1.01–1.26)	0.033	M-H	54.6%	0.138
TT + TC versus CC	1.10(0.96–1.26)	0.161	M-H	22.9%	0.255
TT versus TC + CC	1.59(1.15–2.20)	0.005	M-H	63.2%	0.099
TT versus CC	1.66(0.93–2.99)	0.089	D-L	67.2%	0.081

OR, odds ratio; CI, confidence intervals; I^2^, I-square; M-H, Mantel–Haenszel; D-L, DerSimonian–Laird.

^*^All the three studies were based on MassARRAY platform of Sequenom (without data from the Hong Kong GWAS).

**Table 3:** Genetic interaction analysis between *TBX21* and *IFNG*.

SNP combinations	Test model*	Multiplicative interaction		Additive interaction	
		OR (95% CI)	*P*value	RERI (95% CI)	AP (95% CI)
rs4794067- rs2069705	Codominant model	1.31(1.03–1.66)^a^	0.027		
		1.31(1.03–1.67)^b^	0.026		
	Dominant model	3.04(1.10–8.41)^a^	0.032	0.70(0.33–1.06)^a^	1.18(0.08–2.28)^a^
		2.81(1.00–7.84)^b^	0.049	0.66(0.26–1.07)^b^	1.16(−0.01–2.34)^b^
rs4794067- rs2069718	Codominant model	1.09(0.81–1.46)^a^	0.569		
		1.09(0.81–1.47)^b^	0.567		
	Dominant model	1.79(0.53–6.01)^a^	0.350	0.45(−0.27–1.16)^a^	0.62(−0.53–1.77)^a^
		1.70(0.50–5.81)^b^	0.398	0.42(−0.35–1.18)^b^	0.61(−0.66–1.88)^b^

SNP, single nucleotide polymorphisms; OR, odds ratio; CI, confidence intervals; RERI, the relative excess risk due to interaction; AP, the attributable proportion due to interaction.

^*^The risk allele of rs4794067 and rs2069705 is T, the risk allele of rs2069718 is C.

^a^Genetic interaction analysis without adjusting for age and sex.

^b^Genetic interaction analysis with adjusting for age and sex.
